# The impact of a telephone hotline on suicide attempts and self-injurious behaviors in patients with borderline personality disorder

**DOI:** 10.3389/fpsyt.2023.1288195

**Published:** 2024-01-04

**Authors:** Alice Buronfosse, Marion Robin, Mario Speranza, Philibert Duriez, Jérôme Silva, Maurice Corcos, Fabienne Perdereau, Nadia Younes, Lionel Cailhol, Philip Gorwood, Alexandra Pham-Scottez

**Affiliations:** ^1^Centre Psychiatrique d’Orientation et d’Accueil, GHU Paris Psychiatrie et Neurosciences, Paris, France; ^2^Service de psychiatrie de l’adolescent et du jeune adulte, Institut Mutualiste Montsouris, Paris, France; ^3^Université Versailles Saint-Quentin, Université Paris-Saclay, Inserm U1018, CESP, Team DevPsy, Villejuif, France; ^4^Service Universitaire de Psychiatrie de l’Enfant et de l’Adolescent, Centre Hospitalier de Versailles, Versailles, France; ^5^Clinique des Maladies Mentales et de l’Encéphale, GHU Paris Psychiatrie et Neurosciences, Paris, France; ^6^Université Paris Cité, Institute of Psychiatry and Neuroscience of Paris (IPNP), Inserm, Paris, France; ^7^Clinique Fondation Santé des Etudiants de France, Paris, France; ^8^Service Hospitalo-Universitaire de Psychiatrie de l'Adulte et d'Addictologie, Centre Hospitalier de Versailles, Le Chesnay, France; ^9^Department of Psychiatry, Institut Universitaire de Santé Mentale de Montréal, CIUSSS of East Montreal, University of Montreal, Montreal, QC, Canada

**Keywords:** borderline personality disorder, hotline, suicide attempt, self-injurious behavior, self-harm

## Abstract

**Background:**

Borderline personality disorder is often associated with self-injurious behaviors that cause personal suffering, family distress, and substantial medical costs. Mental health hotlines exist in many countries and have been shown to be effective in some contexts, but none have been specifically designed for borderline patients. The aim of the present study is to evaluate the impact of a 24/7 hotline dedicated to patients with borderline personality disorder on suicide attempts and self-injurious behaviors.

**Methods:**

We conducted a single-blind, multicenter (9 French centers) clinical trial with stratified randomization (by age, sex and center). Patients (*N* = 315) with a diagnosis of borderline personality disorder (according to the SIDP-IV) were randomized into two groups with or without access to the hotline in addition to treatment as usual. The number of suicide attempts and self-injurious behaviors in each group within 12 month were analyzed in the “per protocol” population (Student’s *t*-tests, 5% significance threshold), adjusting for possible confounders in a multivariate analysis (using Poisson regression). The percentage of patients with suicide attempts and with self-injurious behaviors (and other percentages) were analyzed in the per protocol population (χ^2^-tests or exact Fischer tests, 5% significance threshold).

**Results:**

The mean number of suicide attempts was 3 times lower in the hotline group (0.41 vs. 1.18, *p* = 0.005) and the mean number of self-injurious behaviors was 9 times lower (0.90 vs. 9.5, *p* = 0.006). Multivariate analysis confirmed the effectiveness of the hotline in reducing suicide attempts and self-harm.

**Conclusion:**

This study supports the effectiveness of hotlines in reducing self-aggressive behavior in patients with borderline personality disorder. Such support is easy to use, cheap and flexible, and therefore easy to implement on a large scale.

## Introduction

Borderline personality disorder (BPD) is characterized by a pervasive pattern of instability in affect regulation, impulse control, interpersonal relationships, and self-image. Clinical signs of the disorder include emotional dysregulation, impulsive aggression, repeated self-injury, and chronic suicidal tendencies. This disorder is common, its prevalence ranges from 0.7 to 5.9% in the general population (1, 2), reaches 10% in psychiatric outpatients (3), and peaks at 15–25% in psychiatric inpatients (4).

Self-harm behaviors, including suicide attempts (SA), behaviors with the aim of killing oneself without succeeding, and self-injurious behaviors (SIB), behaviors with the aim of physically injuring oneself without endangering one’s life, represent one of the major challenges in the treatment of patients with BPD, as 50–80% of borderline patients self-mutilate at least once in their lifetime (5) and 84% of borderline patients commit at least one SA (6). The repetitive nature of SA and SIB is common in BPD and is related to the impulsivity and emotional instability that characterize this disorder. It is estimated that about 44% of borderline patients have more than five lifetime SA. The risk of recurrence is long-term, as aging is a risk factor (5). 41% of SIB are performed more than 50 times per patient (5). The main complications are impaired quality of life, significant need for medical care (7) and death by suicide, which affects 3–10% of BPD patients (8).

Psychotherapeutic interventions have been developed for BPD (9). Dialectical Behavior Therapy (DBT) is the first therapy to be used specifically for multisuicidal BPD patients and has the strongest evidence of efficacy (10). DBT is a structured outpatient treatment developed by Marsha Linehan, based on cognitive-behavioral principles. DBT is structured into 4 components, including skills training group, individual psychotherapy, telephone consultation, and therapist consultation team (11). These components work together to teach behavioral skills that target common symptoms of BPD, including an unstable sense of self, chaotic relationships, fear of abandonment, emotional lability, and impulsivity such as self-injurious behaviors. The skills include mindfulness, distress tolerance, emotion regulation and interpersonal effectiveness. “Dialectical” refers to the integration of both acceptance and change as necessities for improvement. DBT aims to address the symptoms of BPD by replacing maladaptive behaviors with healthier coping skills, such as mindfulness, distress tolerance, emotion regulation and interpersonal effectiveness.

The DBT hotline is recommended to be available 24 h a day, to allow the patient to seek help in case of risk of self-aggressive behavior, to remind them of acquired skill and to maintain the therapeutic link (11). The hotline provides real-time help, in line with the main principles of DBT. The DBT-trained responder helps borderline patients to better regulate their emotions and increase their tolerance of distress, when they need it, and in a personalized way. According to the theoretical model of DBT, SIB and SA occur in response to emotional pain or intense stress and may act as emotional regulators. SIB and SA cause suffering to patients and their families, increase the need for medical and psychiatric care, and can be life-threatening. However, the specific effectiveness of this DBT hotline on SA or SIB has not yet been demonstrated.

There are approximately 800,000 suicide deaths per year worldwide (12), and various preventive measures have been developed, including restricting access to suicide means, awareness programs, drug treatments (such as clozapine and lithium) and psychotherapies (13). Hotlines are a mean of suicide prevention, with the advantage of respecting the anonymity of the patient and being accessible at any time to provide remote care. Since the first Samaritan center in London in 1953 (14), centers have been set up to enable people from the general population to contact a hotline whenever they feel at risk of self-aggressive behavior. Several studies in different countries have shown that this device can be beneficial in reducing suicidal ideation and SA (15–18). Other studies have examined the characteristics of hotline users and the modalities of calls (19, 20). However, there is a gap in the research in this area, and it is very surprising to note that no study has looked specifically at the effect of a telephone hotline on borderline patients, who are at very high risk of attempting suicide. There is therefore an urgent need to set up a tailored hotline for borderline patients, and to evaluate its effectiveness.

The aim of our study is to demonstrate the impact of a 24/7 telephone hotline, in line with the principles of DBT regarding telephone coaching (11), with responders trained to DBT, on SIB and SA in patients with BPD. Our study compares two groups of patients: the study group with access to a hotline in addition to treatment as usual (TAU), and the control group with TAU only. We hypothesized that patients with BPD who received hotline support plus TAU would have less SA and SIB than patients who received TAU only.

## Materials and methods

### Aims of the study

Our study is a single-blind, multicenter, randomized trial. The primary objective of our study is to demonstrate the effectiveness of the proposed hotline by showing a significant reduction in the number of SA and SIB in the study group compared to the control group. The secondary objective is to determine which subpopulation would benefit most from this device according to gender, treatment center, presence of an Axis I comorbidity and average number of comorbid Axis II disorders. Proof of the effectiveness of a telephone hotline in reducing the number of suicide attempts by borderline patients would make it possible to consider its implementation on a large scale.

### Study population

Clinical recruitment was carried out within a research network specializing in BPD (21). Borderline patients were included in nine French centers (detailed in the Acknowledgements section) from four cities in France (Caen, Paris -six centers-, Toulouse and Versailles).

Inclusion criteria were (1) to be an inpatient or outpatient at one of the nine centers, (2) to be between 18 and 60 years old, (3) to have a diagnosis of BPD according to the SIDP-IV (22), (4) to be fluent in French, and (5) to be registered with the Sécurité Sociale (the French national health insurance system). Patients with other Axis I diagnoses (except schizophrenia) and other Axis II comorbid diagnoses could be included. Exclusion criteria were (1) the presence of a serious somatic illness likely to affect vital prognosis within 1 year, (2) inability to respond to assessments, (3) being placed under guardianship or curatorship, and (4) concurrent participation in another medical research study.

### Assessment

Assessment instruments at enrolment (T1) included:

- the SIDP-IV (Structured Interview for the Diagnosis of DSM-IV Personality) (22), to diagnose BPD and other DSM-IV personality disorders. The SIDP-IV indicates the presence of BPD if at least 5 out of 9 BPD items score 2 or 3 (on a scale of 0 to 3). The BPD dimensional score is obtained by summing the scores of the 9 items, resulting in a total BPD dimensional score ranging from 0 to 27.
- the SCID-I (Structured Clinical Interview for DSM-IV Axis I Disorders) (23) to diagnose the presence of Axis I disorders.
- a self-administered questionnaire including: socio-demographic information (sex, age, family situation, educational level, occupation), psychiatric history (medical follow-up, psychotherapy, hospitalizations, SA, SIB). The self-administered questionnaire at baseline was used to collect the history of SIB and SA.

Clinical psychologists involved in the protocol were initially trained to administer the SIDP-IV. Interrater reliability was assessed by scoring videotapes of 10 patients per psychologist. The interrater reliability for the SIDP-IV was satisfactory: the kappa coefficient was 0.84 for the presence/absence of BPD and 0.95 for the interclass correlation for the BPD dimensional score.

### Study procedure

Clinicians at the nine recruitment centers offered all patients with a BPD the opportunity to participate in the study. During this intake visit, the study was explained to the patient and the inclusion and exclusion criteria were reviewed (SIDP-IV, SCID-I). If a patient agreed to participate in the study, he or she signed an informed consent form and completed the self-administered anamnestic and sociodemographic questionnaire.

Patients were then randomized (with stratification based on age, sex, center and balanced in blocks of six) to one of the two groups (hotline + TAU versus TAU only). Randomization was centralized in a specialized company, computerized and accessible via a voice server. After the inclusion visit (T1), patients received access to the hotline plus TAU (study group) or TAU only (control group) for a period of 12 months.

TAU is a monthly follow-up by the borderline patient’s referring psychiatrist, with parallel weekly follow-up by a psychologist, in psychotherapy. In the TAU, regardless of the group (with or without telephone permanence), no patient was followed-up with DBT.

There were 162 patients in the study group and 153 in the control group. Patients who benefited from the hotline were given a card with the telephone number of the hotline and instructions to call “as soon as he/she feels an inner tension or anxiety that could lead to SA or SIB.” All patients were made anonymous in the database. At the exit visit, 1 year later (T2), we counted the total number of SA and SIB for each patient in a daily logbook completed by the patient.

### Hotline

The hotline was set up for the study and managed by seven clinical psychologists trained in DBT (24). All psychologists had previous theoretical training in DBT and at least 12 months of supervised practice. Any patient in the study group could call the hotline 24 h a day, 7 days a week, in the event of suicidal ideation or perceived risk of SIB. Throughout the study, monthly meetings and supervision were organized by the coordinator of the study (APS) with all the psychologists to discuss any difficult clinical situations that had occurred during the previous month. A large part of their telephone responses was based on the borderline patient’s acquisition of specific skills (in case of distress or acute crisis).

### Ethical aspects

The Sainte-Anne Hospital Centre was the sponsor of this study. The protocol was approved by the *Comité de Protection des Personnes (CPP) Ile-de-France III*. The declaration of the computerized file of personal data collected for the research was made to the *Commission Nationale Informatique et Libertés (CNIL)* before the start of the study. After verbal and written information, all patients signed a written consent to participate in the study.

### Statistical analysis

The main outcome of interest, the number of SA and SIB per patient within 12 months, were analyzed in the “per protocol” population (based on patients who were successfully followed-up) using a Student t-test with a significance threshold of 5%. Secondary endpoints, the percentage of patients with SA and with SIB within 12 months, were analyzed in the per protocol population with a significance threshold of 5%. The statistical test used to compare the percentages of subjects in the two groups was a χ^2^-test or an exact Fischer test.

Multivariate analyses were performed with the following explanatory variables: treatment group, sex, axis I comorbidity, treatment center and presence of an eating disorder. Given the discrete and positive nature of our dependent variables SA and SIB numbers, we used Poisson regressions to model these count data. Coefficients are reported as expected adjustments in the outcome, on a logarithmic scale, for each one-unit change in the covariate (i.e., if β1 is the coefficient, e^β1 is the rate ratio, which means e^β1% change in SA rate).

The socio-demographic characteristics and anamnestic criteria of the participants were analyzed as percentages and mean values. Comparison between the two groups was performed using a χ^2^-test or an exact Fisher test for qualitative data and a Student’s *t-*test or a Wilcoxon test for quantitative data (with a significance threshold of 5%). The same procedure was used to compare subjects who remained in the study with those who were lost to follow-up.

All analyses were performed using R software version 3.5.2 and glm package.

## Results

### Patient sample

A total of 315 patients with BPD were recruited. Females predominated (91%) and the population was young, with a mean age of 27.9 years (*SD* = 8). There was a high proportion of obsessive-compulsive personality disorder (37.3%) and avoidant personality disorder (35.6%). There was also a high proportion of eating disorders, with 56.3% of patients having anorexia nervosa and 53.2% having bulimia nervosa. Sociodemographic and anamnestic characteristics of the study population are summarized in Table 1. The results of the SCID-I and SIDP-IV (number of patients and percentages of patients with at least one Axis I disorder or with at least one other personality disorder) are summarized in Table 2.

**Table 1:** Sociodemographic and anamnestic characteristics of a sample of 315 patients with borderline personality disorder.

	%	N	Mean	SD
Sociodemographic characteristics				
Gender (female) Age Marital status Married/PACS^a^/cohabitating Single Divorced Adopted Living arrangements Living alone With parents/other family members With spouse/companion With flatmate Foster home or host family In study care, therapeutic home In college, student residence Other place Having child(ren) Level of education No diploma College patent CAP^b^/BEP^c^ General or professional bachelor BTS^d^, DUT^e^, DEUG^f^ Post-graduate diploma Other qualification Student Current occupation/last job Individual farmers artisans and traders Managerial and professional occupations Intermediate occupations Employees Blue-collar workers Unemployed	91.0 21.3 74.9 2.4 3.8 36.7 24.8 21.5 4.4 1.5 1.0 1.5 0.5 13.7 3.3 8.6 9.6 30.6 15.8 25.4 6.7 39.6 0.0 2.6 18.5 24.1 31.8 5.6 17.4	192 48 158 5 8 77 52 44 9 3 2 3 1 29 7 18 20 64 30 53 14 84 0 5 36 47 62 11 34	27.9	8
Anamnestic characteristics				
History of psychiatric treatment	94.8	199		
History of psychotherapy	69.9	144		
History of hospitalization	96.2	196		
History of self-harm	80.0	164		
History of suicide attempts	82.6	171		
Mean number of lifetime suicide attempts			3.5	6.3

^a^Alternative to marriage in France. ^b^Certificate of professional competence. ^c^Professional qualification. ^d^Higher technical diploma. ^e^Technical university degree. ^f^Bachelor’s degree.

**Table 2:** Percentage and number of patients with a SCID-I (Axis I) diagnoses or SIDP-IV (Axis II) diagnoses in a sample of 315 patients with borderline personality disorder.

	%	N	Mean	SD
SCID-I diagnoses (current or previous episode)				
Schizophrenia Mood disorders Bipolar 1 disorder Bipolar 2 disorder Major depressive episode Recurrent depressive disorder Anxiety disorders Panic disorder with agoraphobia Panic disorder without agoraphobia Generalized anxiety disorder Agoraphobia without panic disorder Social phobia Specific phobia Post-traumatic stress disorder Obsessive-compulsive disorder Eating disorders Anorexia nervosa Bulimia nervosa Somatoform disorder Hypochondria Substance use disorders Alcohol use Amphetamines Cannabis Cocaine Hallucinogens Inhalants Opioids Phencyclidine Tranquilisers/hypnotics/anxiolytics Other substances	0 8.7 4.8 23.1 69.2 26.9 23.1 46.8 8.0 23.7 34.9 41.8 27.2 56.3 53.2 5.8 4.5 49.4 6.1 29.2 10.9 6.4 0.6 4.2 0.3 18.6 4.5	0 27 15 72 216 84 2 146 25 4 109 130 85 175 166 18 14 154 19 91 34 20 2 13 1 58 14		
SIDP-IV diagnoses				
Total SIDP-IV Borderline score			17.5	3.9
Mean number of personality disorders (including borderline personality disorder)			2.2	1.1
Cluster A				
Paranoid	8.4	26		
Schizoid	1.0	3		
Schizotypal	2.3	7		
Cluster B				
Antisocial	8.7	27		
Borderline	100.0	315		
Histrionic	4.2	13		
Narcissistic	3.8	12		
Cluster C				
Avoidant	35.6	112		
Dependent	23.4	72		
Obsessive-compulsive	37.3	117		

The study and control groups were comparable in terms of sociodemographic, anamnestic and psychiatric comorbidity characteristics. There were no significant differences at enrolment (T1) between the study group and the control group with regard to:

- lifetime history of SA (82.1% of the study group, 93% of the control group, *p* = 0.86).
- lifetime history of SIB (83.9% of the study group, 76.8% of the control group, *p* = 0.22).
- mean number of lifetime SA (mean number of SA:2.96 in the study group and 3.97 in the control group, *p* = 0.27).

### Follow-up

Two people died by suicide during the study, one in each group (0.6%).

At the end of follow-up, there were 97 patients in the study group and 85 patients in the control group. The percentage of patients lost to follow-up is high, because borderline patients often have a fairly unstable lifestyle, and change homes (especially in urban areas like our patients) and change telephone numbers quite frequently. However, we only had these two means, their telephone number and the postal route, to contact patients again after a year. The percentage of participants lost to follow-up was similar in the two groups (Table 3): 131 participants were lost to follow-up (41.6% of the sample), 64 in the study group (39.5%) and 67 in the control group (43.8%) (χ^2^ = 0.595, df = 1, *p* = 0.441).

**Table 3:** Comparison of suicide attempts, self-injurious behaviors and use of care in the hotline group and the control group (during the 12 months of the intervention).

	Hotline group *N* = 97		Control group *N* = 85		*t*	df	*p*
	Mean	SD	Mean	SD			
Number of suicide attempts (SA) per patient	0.412	0.787	1.176	2.346	2.865	100.54	0.005
Number of self-injurious behaviors (SIB) per patient	0.897	1.623	9.5	29.996	2.625	83.42	0.010
Number of hospital admissions per patient	0.639	1.301	0.847	1.555	−1.133	164.9	0.259
Number of emergency department visits per patient	0.505	1.715	0.600	1.104	0.449	165.89	0.654

	Hotline group *N* = 97		Control group *N* = 85		χ^2^	df	*p*
	N	%	N	%			
Patients with at least one SA	28	28.87	26	30.60	0.064	1	0.800
Patients with at least one SIB	38	39.18	40	47.06	1.150	1	0.284
Lost to follow-up	64	65.98	67	78.82	0.595	1	0.441

No differences were found in sociodemographic and anamnestic characteristics and scores on clinician-based questionnaires between patients who remained in the study for 1 year and those who were lost to follow-up, except that there were more employees (40.8%/26.6%, χ^2^ = 4.217, df = 1, *p* = 0.04) and lower educational attainment (16.2%/6.1%, χ^2^ = 5.579, df = 1, *p* = 0.018) in the lost to follow-up group than in the group of patients who remained in the study (Supplementary Table S1).

### Suicide attempts

The primary endpoint, the average number of SA per patient during the 12 months of the intervention, is significantly lower in the study group (0.41 SA/patient) than in the control group (1.18 SA/patient). Nevertheless, we found similar percentages of patients with at least one SA between the two groups during the study year, with 28.9% of the study group having at least one SA compared to 30.6% of the control group (Table 3). Access to the hotline did not reduce the number of patients who committed at least one SA.

### Self-injurious behaviours

The analysis of the primary endpoint for SIB during the 12 months of the intervention shows a lower frequency of SIB in the study group (0.90 SIB per patient) compared to the control group (9.5 SIB per patient, t = 2.625, df = 83.42, *p* = 0.010). Regarding SIB, we observed no difference in the proportion of patients who self-harmed at least once during the study, 39.2% in the study group and 47.1% in the control group (Table 3).

We then used a multivariate analysis (Poisson regression) to assess if the efficacy of the hotline in supporting the reduction of the risk of SA and SIB was still significant after controlling for different potential confounders (center effect, gender balance, and main psychiatric comorbidities). There is indeed significantly more SA risk in patients with major depressive disorder (RR = 2.099; *p* < 0.05) and in patients with substance use disorders (RR = 2.67; *p* < 0.01) (Table 4). Analysis shows that the hotline reduces both SA risk by 70% (RR = 0.316; *p* < 0.001) and SIB risk by 83% (RR = 0.170; *p* < 0.05), after adjusting for gender, comorbidity with depressive, addictive or eating disorders and taking into account a possible center effect.

**Table 4:** Poisson regression.

	Number of SA per patient		Number of SIB per patient	
	RC	RR (e^RC)	RC	RR (e^RC)
Hotline	−1.152	0.316***	−1.774	0.170*
Gender	0.553	1.740	1.663	5.280
Centre	0.016	1.017	−0.252	0.776
Depression	0.741	2.099*	1,0.595	4.932
Drug addiction	0.982	2.670***	0.404	1.499
Eating disorders	0.334	1.397	−2.377	0.093

*p < 0.05, **p < 0.01, ***p < 0.001. RC, regression coefficient.

### Hotline use

The hotline evaluation completed by patients at the end of the study indicates low use of the hotline (34% of callers in the study group). However, 52% of patients reported that they had thought about calling the hotline at least once in the year during which they had access to the hotline. Thinking about calling the hotline can help the patient to think better about the crisis situation they are going through (by making them anticipate what they will say if they call). In some cases, this may be enough to reduce internal tension, so that the patient actually no longer needs to call. Seventy five percent of the patients said that the availability of the hotline helped them to avoid SA or SIB. In some cases, the availability of the hotline allowed them to reduce their feeling of impasse and abandonment.

### Other variables

There were no differences between the study and control groups in the mean number of hospital admissions and the mean number of emergency department visits (Table 3). The hotline therefore did not make it possible to reduce the use of care by borderline patients.

## Discussion

By proposing to analyze the added value of a hotline specifically dedicated to reducing the risk of SIB and SA for 1 year, we found that such a hotline is effective, reducing the risk of SA by 2.88 and of SIB by 10.56 in terms of numbers of observed events during the time of the study. Clinically, the reduction of SA occurrences should lead to an increase in safety and life expectancy of borderline patients, since the repetition of SA often precedes a completed suicide. The reduction of SIB occurrences should help limit the suffering of patients, and the feeling of helplessness of loved ones and caregivers. On the other hand, the proportion of suicidal or self-harming patients (at least one instance of SA/SIB) was not significantly different between the study and control groups, leading to the conclusion that the impact of such a hotline is more quantitative (reducing their number) than qualitative (eliminating them). The quantitative reduction of self-harm probably improve the general well-being of borderline patients, therefore contributing to their recovery. It will also ensure that psychiatrists and nurses do not become discouraged in the complex task of taking care of patients with borderline personality disorder.

We can assume that even if access to the hotline was effective in preventing recurrences, the patient needed to have an experience (a call) in order to benefit from its advantages. Once the contact was made, the hotline could be considered as an effective support. It can be assumed that the feeling of shame and the fear of disturbing others may have hindered recourse to the hotline. In addition, some patients may have forgotten their participation in the study and the possibility of using the hotline. The study did not provide for reminders between the inclusion visit and the exit visit. Regular reminders of the existence of this hotline, by treating psychiatrists, nursing teams, psychologists and patients’ entourage, should greatly increase the percentage of use of the hotline by borderline patients. We can also imagine regular and proactive reminders from the hotline responders for patients not using it, encouraging them to use this opportunity more frequently and quickly.

Our sample is predominantly young and female, with a high number of Axis II comorbidities, in line with what is usually described in the existing literature (24–29). The mean BPD dimensional score obtained in the SIDP-IV corresponds to a medium severity of BPD, which is comparable to that found in another study (30). The second most common comorbid personality disorder in our population is obsessive-compulsive personality disorder. This may seem surprising given the semiological differences between borderline and obsessive-compulsive personality disorders. However, it can be explained by the high proportion of patients also presenting with an eating disorder such as anorexia nervosa or bulimia nervosa, which was particularly common in the largest recruitment center (CMME). There is a frequent association in the literature between eating disorders and obsessive-compulsive personality disorder, especially anorexia nervosa (31), which may explain the high prevalence of this personality disorder in our population. In terms of substance use disorders, alcohol use disorder was the most common, similar to the literature (32), and other use disorders were present in similar proportions.

It is interesting to see how the interactions with the psychologist were able to reduce self-harm. Establishing communication may have delayed patients’ feelings of abandonment and emptiness long enough for the crisis to pass. This makes it possible to apply Marsha Linehan’s theory by allowing the caller to mobilize skills of distress tolerance and emotion regulation. The use of a third party thus makes it possible to better manage the emotions arising from these moments of intense stress. It can also help to mentalize these situations, allowing a better awareness of the triggers of these crises and to anticipate future events.

In our sample, SA was more common in patients with a history of depression and/or substance use disorders, which is consistent with previous literature identifying these two variables as indicators of increased risk of SA in borderline patients (5, 6). It is therefore interesting that multivariate analyses controlling for these two comorbid disorders still indicate that the hotline was significantly protective. From a clinical point of view, this result could mean that subpopulations like those with a history of depression or of substance abuse, at high risk of SA and SIB, could particularly benefit from such a hotline process, whereas usually the preventive effect of hotlines decreases in patients with higher severity (33) and/or more frequent psychiatric comorbidity (34).

The main limitation of this study is the number of people lost to follow-up (41.6% of the sample). We expected a significant proportion of loss to follow-up, as BPD is associated with frequent withdrawal from care and medical nomadism. However, data in the literature report a lower percentage of dropouts, ranging from 9.6 to 26% (35–37). This difference may be explained by the nature of some recruitment centers, including emergency centers, which do not follow up patients. It should also be noted that the French health care system does not force patients to maintain a permanent relationship with a consistent therapeutic team, but rather allows patients to choose and change practitioners as they wish. We expected a high rate of loss to follow-up, but were very surprised that it largely exceeded 20% (the expected attrition rate). This high rate limits the representativeness of our results and the reliability and generalization of them. However, it is important to note that the number of patients lost to follow-up was not different in the two groups, and that the drop-out patients were comparable to the follow-up patients. Large attrition rates have lower impact on per protocol analyses (used herein) compared to intention to treat approaches, but the consequence is that the conclusions concerning the efficacy detected in our study has to be limited to patients using the hotline support the full year.

In conclusion, our study has shown the effectiveness of this hotline in reducing the recurrence of self-harm, with the average number of SA per patient being 3 times lower in the hotline group and more than 9 times lower for SIB. The effect of such a hotline could be improved by regular reminders of its existence (38), especially to prevent the first act of self-harm. Future studies are needed to test this hypothesis. Our study has important public health implications, as the implementation of this type of hotline dedicated to borderline patients is easy and feasible on a large scale, is efficient in reducing the number of SA and SIB, and the affordability is an additional argument.

## Data availability statement

The raw data supporting the conclusions of this article will be made available by the authors, without undue reservation.

## Ethics statement

The studies involving humans were approved by Comité de Protection des Personnes Ile-de-France III. The studies were conducted in accordance with the local legislation and institutional requirements. The participants provided their written informed consent to participate in this study.

## Author contributions

AB: Writing – original draft. MR: Investigation, Writing – review & editing. MS: Conceptualization, Writing – review & editing. PD: Writing – review & editing. JS: Methodology, Writing – review & editing. MC: Supervision, Writing – review & editing. FP: Investigation, Writing – review & editing. NY: Investigation, Writing – review & editing. LC: Investigation, Writing – review & editing. PG: Investigation, Writing – review & editing. AP-S: Conceptualization, Funding acquisition, Supervision, Writing – review & editing.
