# Supplementary material for: The impact of a telephone hotline on suicide attempts and self-injurious behaviors in patients with borderline personality disorder
Source: Front Psychiatry. 2024 Jan 4;14:1288195. doi: 10.3389/fpsyt.2023.1288195 (PMC10794764; doi:10.3389/fpsyt.2023.1288195)
Supplement: Supplementary file 1 [file Table_5.docx]

Supplementary data : comparison between patients who remained into the study and patients lost to follow up

|  | **Remained patients (N=182)** | | **Lost to follow up**  **(N=131)** | | **p** |
| --- | --- | --- | --- | --- | --- |
|  | **%** | **mean** | **%** | **mean** |  |
| Sex (women)  Age  Marital status  Single  Married/ PACS/in cohabitation  Divorced  Level of study  No diploma  BEPC  CAP or BEP  General or professional bachelor  BTS, DUT, DEUG  Graduate diploma  Other  Current occupation/last job  Individual farmers  Craftsmen, merchants, business leaders  Managerial and professional occupations  Intermediate professions  Employees  Workers  Unemployed  Psychiatric care  History of psychiatric follow up  Current psychiatric follow up  History of hospitalisation  Mean number of hospitalisations  Emergency room  Mean number of emergency room  Self-harm  History of SA  Mean number of SA  History of SIB  1 to 5 times  > 5 times  Axis I and Axis II  Mean BORDL SIDP-IV score  Mean number of Axis I diagnoses  Mean number of Axis II diagnoses | 90.4  72.1  24.4  2.9  1.5 9.1 6.1 29.5 16.7 28.8 8.3  0 2.4 20.2 27.4 26.6 7.3 16.1  94.9 69.1 92.5  71.9   83  81.3 26.9 54.5 | 27.8                          4.5  3.0  3.2  17.2  5.9  2.2 | 92.1  78.9  19.7  1.3  6.8  8.1  16.2  33.8  10.8  20.3  4.1  0  2.8  15.5  18.3  40.8  2.8  19.7  92.1  66.2  90.8  79.7  81.9  77.5  33.8  43.7 | 28.3  4.4  4.7  4.1  18.0  5.9  2.3 | 0.673  0.753  0.307  0.434  0.656  0.101  0.811 **0.018**  0.528  0.253  0.180  0.387  1  0.419  0.598  **0.040**  0.334  0.525  0.552  0.667  0.667  0.925  0.118  0.239  0.854  0.415  0.509  0.299  0.141  0.072  0.893  0.308 |
